# onsite: An Integrated Framework for Phosphosite Localization and False Localization Rate Estimation

**DOI:** 10.1021/acs.jproteome.6c00570

**Published:** 2026-10-10

**Authors:** Qi-Xuan Yue, Zhongchun Wei, Chengxin Dai, Mingze Bai, Yasset Perez-Riverol, Timo Sachsenberg

**Affiliations:** † Chongqing Key Laboratory of Big Data for Bio Intelligence, 12419 Chongqing University of Posts and Telecommunications, 400065 Chongqing, China; ‡ State Key Laboratory of Medical Proteomics, Beijing Proteome Research Center, National Center for Protein Sciences (Beijing), Beijing Institute of Lifeomics, 102206 Beijing, China; § International Academy of Phronesis Medicine (Guangdong), 510320 Guangdong, China; ∥ European Molecular Biology Laboratory, European Bioinformatics Institute, Wellcome Genome Campus, CB10 1SD Cambridge, U.K.; ⊥ Applied Bioinformatics, Department of Computer Science, 9188 University of Tübingen, 72074 Tübingen, Germany; # Institute for Bioinformatics and Medical Informatics, University of Tübingen, 72074 Tübingen, Germany

**Keywords:** phosphoproteomics, post-translational modification, phosphorylation, onsite

## Abstract

With the rapid development of mass spectrometry-based proteomics, the volume of phosphoproteomic data has increased substantially. However, accurate localization of phosphorylation sites and standardized statistical validation remain critical analytical bottlenecks. To address the lack of standardized cross-algorithm evaluation, we introduce onsite, a unified and open-source Python framework. onsite integrates an alanine-decoy strategy to estimate the false localization rate (FLR) across three algorithms: AScore, PhosphoRS, and pyLucXor. This modular architecture efficiently processes large-scale data sets and enables global FLR calculation. Benchmarking on the standard synthetic phosphopeptide data set PXD000138 highlighted distinct interalgorithmic variations. Using the same 5% global FLR threshold, pyLucXor localized the most target sites (28,353). It also reached a high accuracy (91.22%) against the known ground truth, resulting in the largest number of correctly localized sites (25,865). Reanalysis of the highly fractionated, large-scale PXD012255 data set further demonstrated that native integration of onsite into the quantms pipeline enables scalable processing and provides a standardized framework for FLR control in large-scale phosphoproteomics.

## Graphical abstract

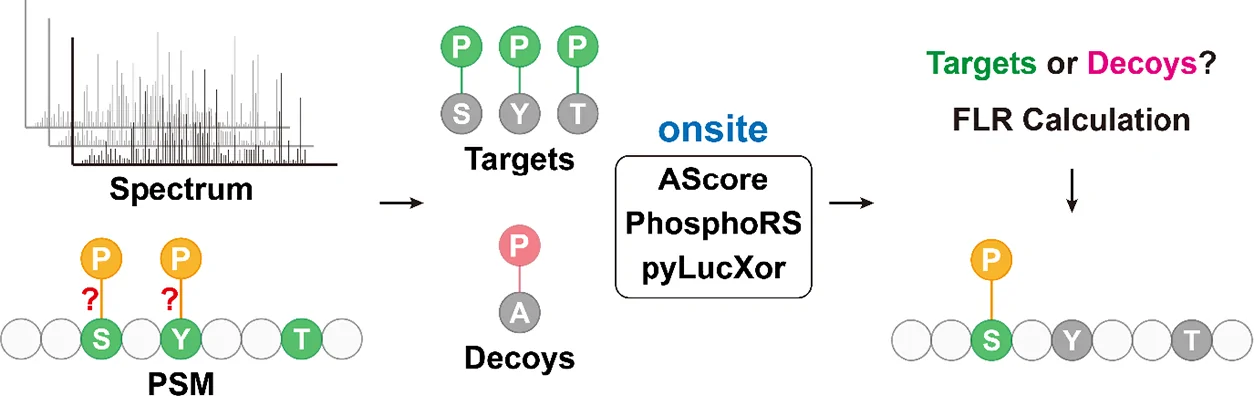

## Introduction

Post-translational modifications (PTMs), particularly phosphorylation, dynamically regulate protein structure, function, and cellular signaling.^1,2^ Phosphorylation occurs primarily on serine (S), threonine (T), and tyrosine (Y) residues.^3^ However, peptides often contain multiple candidate phosphorylation sites, making precise localization of the modification to a specific residue a critical step in phosphoproteomic analysis. Accurate identification of these sites is essential for deciphering the molecular mechanisms governing protein function and regulatory networks.

While standard peptide database searches can match tandem mass spectrometry (MS/MS) spectra to peptide sequences, they are not primarily designed to accurately localize post-translational modifications. They provide confidence estimates for peptide-spectrum matches (PSMs) but typically lack a dedicated assessment of modified site localizations.^4^ This poses a particular challenge for peptides containing multiple candidate residues, where the correct phosphate-group localization remains ambiguous. To resolve positional uncertainties, the need for accurate site localization has driven the development of specialized algorithms. Early pioneering methods utilized probability-based models. For instance, the PTM score^2^ calculates site-specific localization probabilities to classify high-confidence phosphosites and was subsequently integrated into MaxQuant. Similarly, the AScore algorithm employs a binomial statistics metric based on site-determining ions to automate localization.^5^ As a computationally less demanding approach, the Mascot Delta Score infers localization confidence from the difference in search-engine scores between the most likely and the next-best phosphorylation-site assignment.^6^ PhosphoRS uses probabilistic scoring to analyze isoforms through a peak extraction technique adaptable to various fragmentation methods.^7^ Because neither probability- nor delta-scoring methods provide direct control of localization error rates at the data set level, LuciPHOr incorporated a modified target–decoy strategy. Using decoy peptides with artificial modifications on noncandidate residues, it enables simultaneous localization and directly estimates the false localization rate (FLR).^4^ Its successor, LuciPHOr2, extends this FLR estimation to generic PTMs with significantly reduced computation time.^8^

To establish a universally comparable metric for FLR estimation, the phospho-Alanine (pAla) decoy strategy was developed.^9^ Unlike methods that depend on algorithm-specific localization scores, pAla introduces decoy site assignments by allowing phosphorylation on alanine residues, enabling direct estimation of localization error rates. Alanine provides a chemically implausible decoy site for conventional phosphoproteomics. However, target (S/T/Y) and decoy (A) residues naturally occur at different background frequencies within the proteome. Consequently, accurate FLR estimation requires statistical calibration to account for differences in residue frequencies. After calibration, the pAla decoy method enables consistent and method-independent estimation of the FLR across data sets and localization algorithms.

We present onsite, an open-source implementation of alanine-decoy-based FLR estimation that is fully compatible with OpenMS^10^ and quantms.^11^ Rather than introducing a new localization model, onsite provides a unified framework for applying the pAla decoy strategy across established localization algorithms, including AScore, pyLucXor (a Python reimplementation of LuciPHOr2), and PhosphoRS. While the core module performs FLR estimation on individual files, dedicated utility scripts enable aggregation of statistics across multiple files facilitating large-scale phosphoproteomics analysis. By integrating pAla-based FLR estimation into modern bioinformatics workflows, onsite provides a common framework for translating algorithm-specific localization scores into universally comparable estimates of data set-wide localization error.

## Experimental Procedures

### Implementation

Built on PyOpenMS, onsite implements a modular pipeline that spans data import, peptide-spectrum matching, site localization, and FLR calculation, accepting input in open formats. To support large-scale analyses, it parallelizes computationally intensive spectrum processing tasks (e.g., evaluating candidate target and alanine-decoy site assignments that need to be evaluated at multiple levels of peak filtering) and aggregates target and decoy observations across files for data set-level FLR estimation. onsite implements the pAla target-decoy approach (TDA), in which alanine serves as a decoy to model the distribution of random mislocalizations. onsite integrates three localization algorithms. The first algorithm, AScore, localizes phosphorylation sites by comparing alternative site assignments and assessing the evidence provided by site-determining fragment ions. Using a binomial probability model, it calculates how strongly the spectrum supports one localization over competing possibilities under different levels of peak filtering. Higher scores indicate a lower probability of incorrect site assignments. The second algorithm, PhosphoRS, evaluates all feasible site localizations within a probabilistic framework and outputs posterior probabilities for individual modification sites. This allows PhosphoRS to provide a more direct and interpretable measure of site-specific localization confidence compared to AScore. Third, pyLucXor introduces a rigorous delta score distribution model.

Finally, a global pAla FLR module calculates and aggregates statistics across multiple files. It excludes unambiguous peptides (where all potential sites are modified) to prevent an artificial underestimation of the global FLR. onsite reports localization results using the standardized ProForma notation,^12^ ensuring interoperability.

### Benchmarking

We evaluated onsite using the synthetic phosphopeptide library data set PXD000138, comprising 96 HCD raw files available through PRIDE. Peptide identifications generated with the quantms workflow were used as input for a standardized comparison of phosphorylation-site localization algorithms.^13^ The complete upstream search configuration for both PXD000138 and PXD012255 - search engine and software versions, protein sequence database, enzyme specificity, precursor and fragment mass tolerances, fixed and variable modifications, and PSM-level error control, together with the quantms versions and commands used - is provided in the Supporting Information (Table S2). The three algorithms (AScore, pyLucXor, and PhosphoRS) implemented in the onsite package were used for the comparative analysis (Table S1).

Data set processing and peptide database search. Raw files from PXD000138 and PXD012255 were processed with quantms versions v1.3.1dev and v1.8.0dev, respectively. PXD000138 was searched using Comet (2023.01 rev. 2) and MS-GF+ (v2023.01.12), whereas PXD012255 was searched using Comet (2025.01 rev. 1) and MS-GF+ (v2024.03.26). Searches were performed against the human reference proteome database (appended with common contaminants and reversed decoy sequences). The cleavage agent was Trypsin/P for PXD000138 and Trypsin for PXD012255, allowing up to 2 missed cleavages. Precursor mass tolerances were set to 5 ppm for PXD000138 and 20 ppm for PXD012255, with a fragment mass tolerance of 0.02 Da for both data sets. For PXD012255, carbamidomethyl (C) was set as a fixed modification, while no fixed modifications were applied for the synthetic PXD000138 data set. Variable modifications included phosphorylation (S/T/Y) and oxidation (M) for both data sets, with the addition of protein N-terminal acetylation for PXD012255, allowing up to 3 variable modifications per peptide. PSM-level error control was performed using Percolator (version 3.05.0 for PXD000138 and 3.06.0 for PXD012255), and target PSMs with q-values below 0.01 were retained. The complete commands and parameters are provided in Table S2. The resulting mzML and idXML files were used as input to onsite.

The baseline represents the phosphopeptidoform assignment reported directly by the search engines (Comet and MS-GF+) and ranked by Percolator q-values. No dedicated postsearch site-localization algorithm was applied. The baseline was subjected to the same identification-level and sequence filters as the onsite methods.

The 40-run HCT116 phosphoproteomics data set PXD012255 was reprocessed with quantms v1.8.0dev using the *onsite_compute_all_scores* option, which executed AScore, PhosphoRS, and pyLucXor within the same workflow. Algorithm-specific pAla statistics were aggregated across all fractions and filtered at a 1% pAla-estimated global FLR. A phosphosite was distinctly defined by its unmodified peptide sequence and modified residue position. Global FLR estimation and 1% threshold filtering were performed independently at the PSM level. Repeated observations of the same localized site across all analytical runs were collapsed into a set of unique sites to determine the final diagnostic yield and algorithmic overlap. The complete search and workflow parameters are provided in Table S2.

Initially, we systematically compared the localization sites predicted by the algorithms under unified, standardized threshold conditions. To ensure an unbiased evaluation and to strictly isolate localization accuracy from sequence identification confounding factors, we implemented a rigorous, uniform filtering pipeline across all algorithms, integrating principles from Ramsbottom et al.^9^ and Camacho et al.^14^

The unified filtering pipeline comprised four sequential steps. First, all decoy peptide-spectrum matches were removed and FDR was controlled by applying a strict PSM-level *q*-value threshold of <0.01. Second, to eliminate background noise, nonsynthetic peptides were excluded, and only peptides that perfectly matched the target sequences within the synthetic seed library were retained. Third, to avoid the additional complexity introduced by co-occurring modifications, multiply phosphorylated peptides were excluded from the benchmark. This resulted in a controlled data set with a single phosphorylation event per PSM. This restriction applies only to the benchmark; onsite itself supports peptides with multiple phosphorylation sites. Finally, unambiguous peptides (i.e., those with no alternative localization options) were discarded, with the strict prerequisite that a reported site be included in the analysis only if it has a usable localization confidence score.

We estimate the FLR according to Camacho et al.^14^ on the sorted site scores:

$$Estimated\;FLR=\frac{\left(\frac{T}{A}\right)\sum_{i=1}^{n}\left(pA_{i}\right)}{n-\sum_{i=1}^{n}\left(pA_{i}\right)}$$

where: *T* and *A* represent the counts of target and decoy amino acids, ∑_*i* = 1_^*n*^(*pA*_*i*_) represents the cumulative number of decoy sites, and *n* represents the rank.

Second, we evaluated localization accuracy against a ground-truth reference derived from the synthetic phosphopeptide library. Known phosphorylation sites reported in the original publication served as the reference set.^13^ After applying the same quality-control criteria described above and filtering results at an estimated global FLR of 5%, site assignments were compared with the ground truth to determine true-positives and false-positives for each method. We calculate the true FLR, using the method proposed by Camacho et al.:^14^

$$True\;FLR=\frac{\sum_{i=1}^{n}\left(incorrect\;p{\mathrm{STY}}_{i}\right)}{\sum_{i=1}^{n}\left(p{\mathrm{STY}}_{i}\right)}$$

Additionally, we evaluated a conventional database search engine as a baseline using the same filtering criteria as the onsite benchmark. Restricting the analysis to single-site phosphopeptides eliminated localization ambiguity, allowing peptide identification scores to be directly interpreted as localization confidence.

### Interoperability with Quantms and Support of HUPO-PSI Standards

By default, quantms performs site localization using pyLucXor with its standard parameter settings (default parameters shown in Table S3 and Table S5). Users can alternatively select AScore or PhosphoRS through the configuration file (both defaulting to a fragment mass tolerance of 0.05 Da) and customize algorithm-specific parameters as needed. Supported input formats include the community-standard HUPO-PSI formats mzML for spectral data (-in) and mzIdentML^15^ for peptide-spectrum matches (-id), together with the OpenMS-native idXML format and the columnar idparquet format (QPX, https://github.com/bigbio/qpx).

As a postsearch processing tool, onsite does not require the upstream search to be performed within OpenMS or quantms and can therefore be used outside this ecosystem. Spectra are provided in mzML format, while the corresponding PSMs can be supplied in supported open identification formats. Users of common search engines and processing environments, such as MaxQuant, FragPipe/MSFragger, Proteome Discoverer, and Mascot, therefore only need to export or convert their search results to one of these supported formats before analysis with onsite. Direct import of engine-specific result formats is currently not supported.

## Results and Discussion

pyLucXor could not process three low-yield files (73, 94, and 96) because they contained too few high-confidence PSMs for model training. To maintain a consistent comparison across methods, these files were excluded from all analyses, despite being processable by AScore and PhosphoRS. Of the 336,993 initial PSMs, 265,648 were common to all three localization tools. After filtering, including the exclusion of unambiguous peptides, approximately 84,100 PSMs were retained for downstream analysis. Figure 1A shows localization performance across a range of global FLR thresholds. AScore and PhosphoRS identify more target phosphorylation sites at low FLRs (e.g., 1%), whereas pyLucXor achieves the highest yield as the FLR threshold becomes less stringent (e.g., 5 and 10%). Furthermore, to provide an objective benchmarking of algorithmic yield at identical error rates, we evaluated the unique true-positive site counts strictly against the empirically calculated True FLR (Figure S2). This orthogonal evaluation confirmed that while the algorithms perform comparably at a stringent 1% True FLR, pyLucXor demonstrates a pronounced advantage in yield at slightly relaxed thresholds (e.g., 5% True FLR).

**Figure 1:**
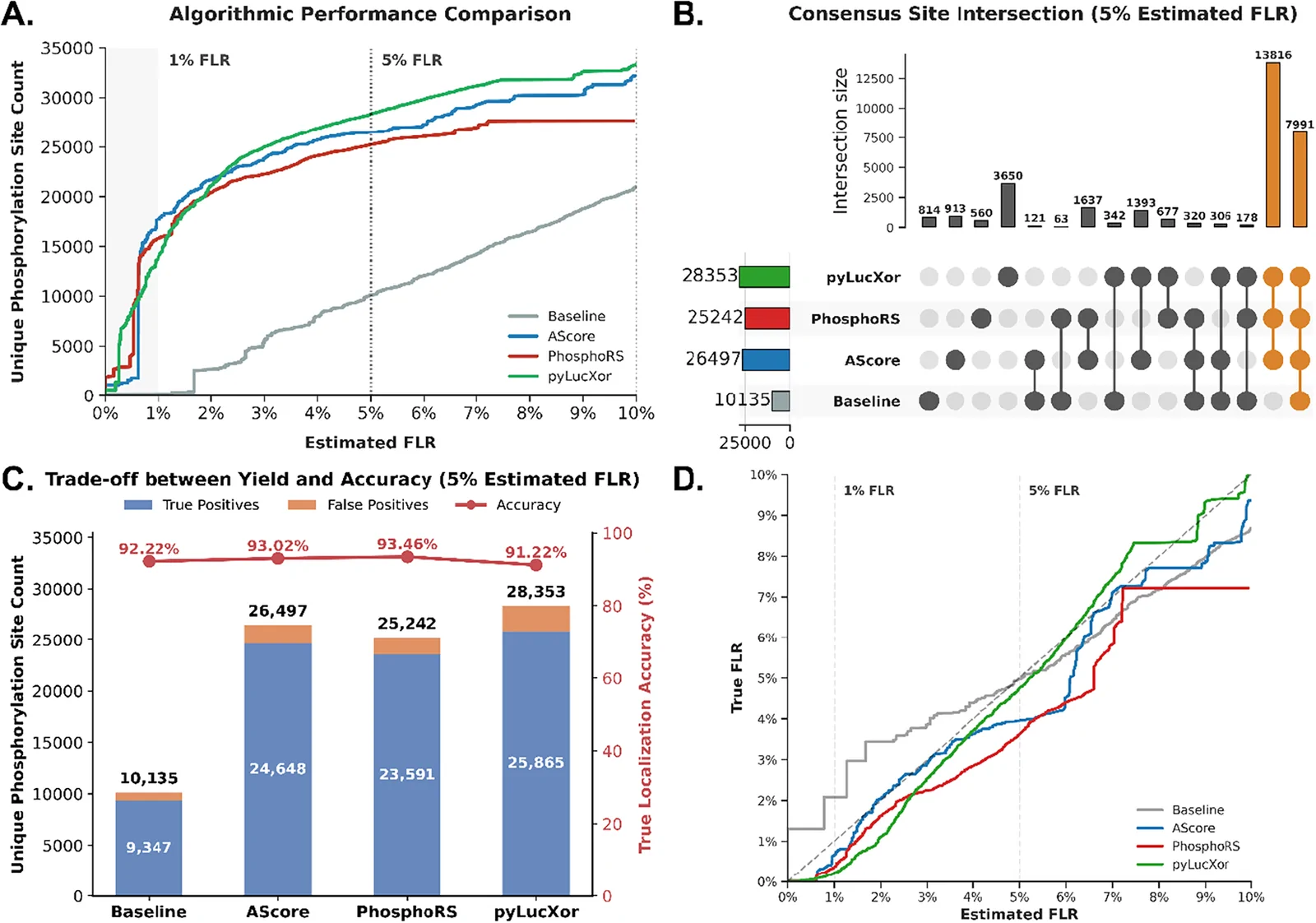
Performance comparison of phosphorylation site localization algorithms under standardized global FLR control. (A) Number of localized target phosphorylation sites reported by the baseline approach, AScore, PhosphoRS, and pyLucXor as a function of estimated global FLR. Dashed lines indicate the 1 and 5% FLR thresholds commonly used for downstream analyses. (B) UpSet plot of target-site calls at 5% pAla-estimated global FLR. The plot contains pyLucXor, PhosphoRS, AScore, and the direct search-engine baseline. Horizontal bars show the total site count for each method, and each vertical bar represents the exclusive intersection defined by the filled circles below it. The 13,816-site bar denotes sites shared by the three dedicated localization algorithms but absent from the baseline; the 7,991-site bar denotes sites shared by all four methods. 21,807 sites were common to AScore, PhosphoRS, and pyLucXor in total. (C) Comparison of localization yield and accuracy at 5% estimated global FLR. Bars show the numbers of true-positive and false-positive site localizations determined using the synthetic peptide ground-truth data set, while the red line indicates localization accuracy. pyLucXor achieved the highest site yield, whereas PhosphoRS achieved the highest localization accuracy. (D) Calibration of estimated versus observed (true) FLR. Perfect calibration corresponds to agreement between estimated and true FLR.

At a global FLR threshold of 5%, pyLucXor identified the largest number of target sites (Table 1), recovering 28,353 localizations compared with 26,497 for AScore and 25,242 for PhosphoRS. To further characterize these results, we examined the overlap and method-specific localizations at the 5% FLR cutoff (Figure 1B). UpSet analysis showed that 13,816 sites were identified by all three algorithms, whereas pyLucXor uniquely localized 3,650 sites not reported by either AScore or PhosphoRS. These results indicate that pyLucXor’s increased yield is largely driven by additional site recoveries beyond the consensus set, while decoy rates remained comparable across methods (6.57–12.57%).

**Table 1:** Localization Performance at Fixed pAla-Estimated Global FLR Thresholds^a^

Algorithm	1% FLR	5% FLR	10% FLR	Total sites/decoys (rate)
pyLucXor	13,892	28,353	33,372	39,762/3,374 (8.49%)
AScore	17,727	26,497	32,195	38,198/2,508 (6.57%)
PhosphoRS	15,819	25,242	27,682	40,068/5,035 (12.57%)
Baseline	180	10,135	21,015	40,155/3,763 (9.37%)

a Numbers of localized target phosphorylation sites identified at 1, 5, and 10% pAla-estimated global FLR.

To assess the balance between localization yield and accuracy, we compared the numbers of true-positive (TP) and false-positive (FP) phosphorylation site localizations at a global FLR threshold of 5% (Figure 1C). The baseline search-engine approach identified 9,347 TP sites, highlighting the limited ability of peptide-level confidence scores to resolve ambiguous modification sites. In contrast, the dedicated localization algorithms substantially increased the number of correctly localized sites, with AScore, PhosphoRS, and pyLucXor identifying 24,648, 23,591, and 25,865 TP sites, respectively. PhosphoRS and AScore achieved the highest localization accuracies (93.46 and 93.02%, respectively), whereas pyLucXor recovered the most correctly localized sites at a marginally lower accuracy, keeping it within about two percentage points of the best-performing method (91.22% versus 93.46%), reflecting a favorable balance between localization sensitivity and accuracy.

We evaluated algorithm calibration by comparing estimated and observed FLRs (Figure 1D). The baseline method underestimated localization error in the low FLR region, whereas PhosphoRS produced conservative FLR estimates throughout the evaluated range. AScore was conservative at low FLRs but became less conservative at higher thresholds. pyLucXor exhibited the closest overall agreement between estimated and observed FLR, although it was slightly conservative below 5% FLR and slightly anticonservative between 5 and 10% FLR before converging near the 10% threshold. Overall, all localization algorithms yielded reasonably well-calibrated results when using the pAla TDA for FLR estimation.

Computational performance was assessed by processing each data set file individually and measuring execution time (Table S4). AScore achieved the highest throughput (227.17 PSMs/s), while pyLucXor was the slowest integrated method (66.36 PSMs/s), consistent with its more extensive statistical modeling. Nevertheless, pyLucXor achieved a processing speed comparable to that of the original Java implementation of LuciPHOr2 (62.1 PSMs/s).

To facilitate adoption and reproducibility, onsite is openly available on GitHub (https://github.com/bigbio/onsite) and distributed via PyPI as a standard Python package. The software provides both an application programming interface (API) and a command-line interface (CLI) for local phosphorylation-site localization analyses.

Furthermore, to ensure platform-independent deployment and reproducible analyses, onsite is distributed through the BioContainers registry. This containerized implementation allows onsite to function as a self-contained module within quantms, a Nextflow-based workflow for quantitative proteomics, thereby integrating multialgorithm phosphorylation-site localization into automated data-processing pipelines. Through Nextflow’s orchestration capabilities, the module can be deployed reproducibly across local, high-performance computing, and cloud environments, supporting scalable reanalysis of large-scale phosphoproteomics data sets.

To demonstrate the scalability and multialgorithmic capabilities of our framework, we reanalyzed a large-scale phosphoproteomics data set (PXD012255) of the HCT116 colon carcinoma cell line^16^ using the quantms workflow. By enabling the onsite_compute_all_scores parameter, AScore, PhosphoRS, and pyLucXor were executed within a single automated workflow. The full reanalysis was performed on a local workstation (Intel Core i9-7900X, 128 GB RAM, Ubuntu 24.04.2 LTS) and completed in 15h 29m 18s.

Following data processing, we assessed algorithm performance across the highly fractionated data set. Despite the data sparsity and variability introduced by capillary zone electrophoresis (CZE) separation, quantms-onsite successfully generated localization scores for all three algorithms across the cohort (Figure 2A). To evaluate the complementarity of the methods, we analyzed the overlap of phosphorylation sites localized at a stringent 1% global FLR after aggregating results across all fractions (Figure 2B). UpSet analysis revealed a substantial consensus set of 4,421 sites shared by all three algorithms, alongside a large number of method-specific localizations, including 3,742 sites uniquely identified by pyLucXor. These results demonstrate that the algorithms provide complementary localization evidence and that combining multiple approaches could increase phosphosite coverage beyond that achieved by any individual method.

**Figure 2:**
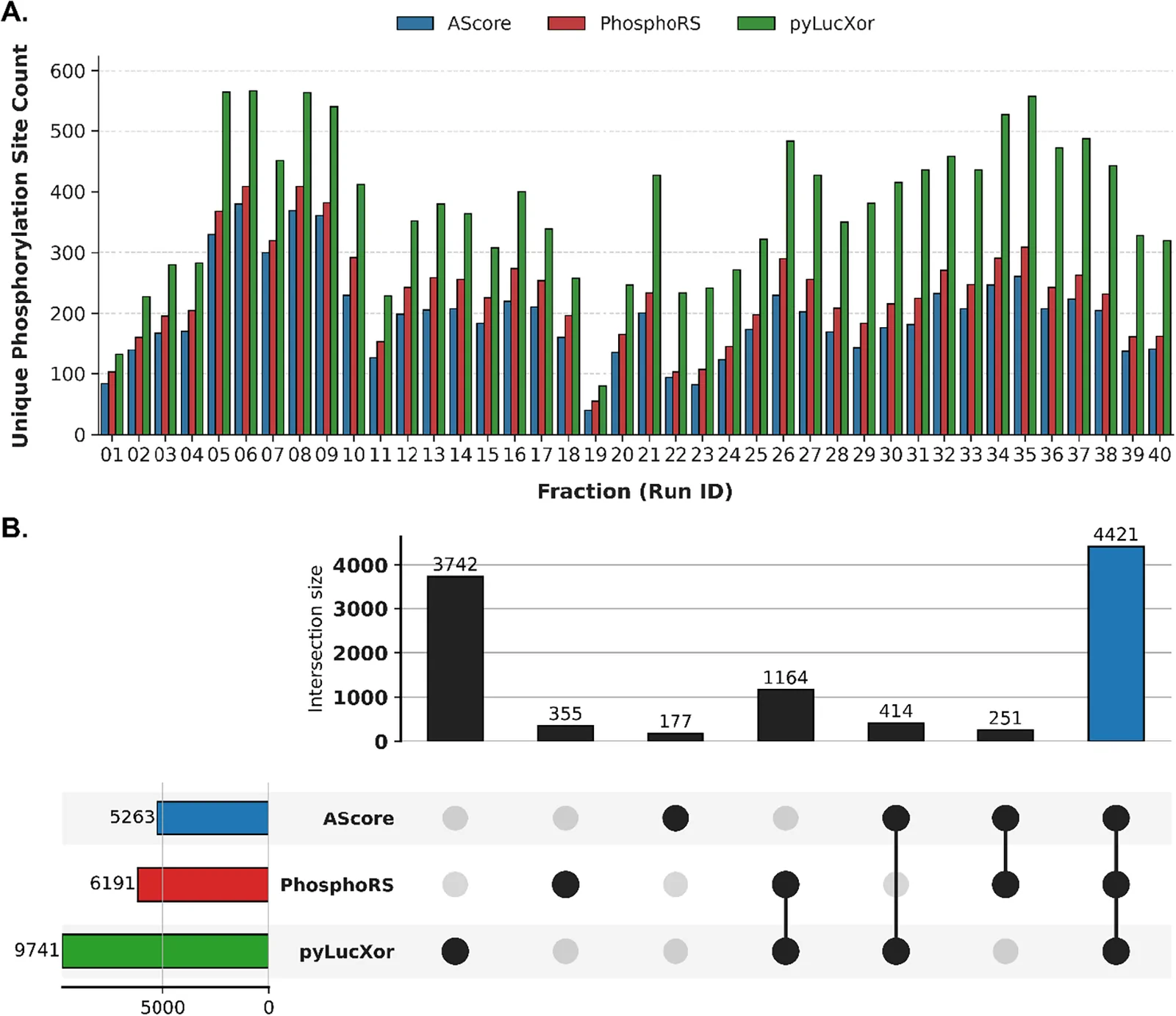
Application of the quantms (onsite) workflow to the HCT116 cell-line data set (PXD012255). (A) Per-file distribution of high-confidence phosphorylation sites across 40 runs, filtered at a 1% global FLR threshold. (B) UpSet plot illustrating the overlap and algorithm-specific high-confidence phosphorylation sites identified by AScore, PhosphoRS, and pyLucXor.

At 1% pAla-estimated global FLR, pyLucXor, PhosphoRS, and AScore reported 9,741, 6,191, and 5,263 sites, respectively. A total of 4,421 sites were shared by all three algorithms, whereas 3,742 sites were reported only by pyLucXor. Comparison with PeptideAtlas (Human Phospho 2025–12) showed that 92.20% (3,450 out of 3,742) of the pyLucXor-exclusive sites were independently validated in the external database, a validation rate similar to the 92.56% (4,092 out of 4,421) observed for the consensus sites. Consensus sites represented highly abundant modifications (averaging 3,108 external observations, 8.36 supporting PSMs, and 1.86 fractions). pyLucXor-specific sites had lower PSM support (averaging 1.91 supporting PSMs across 1.23 fractions) but similar motifs (Figure S1).

Because PXD012255 lacks localization ground truth, we interpreted algorithm-specific sites as candidates with varying levels of supporting evidence, including matches in external phosphosite resources, rather than as confirmed additional sites. Detailed metrics for each algorithm intersection, including external database validation rates and spectral redundancy, are provided in Table S6.

## Conclusion

We present onsite, an open-source Python framework that integrates AScore, PhosphoRS, and pyLucXor within a unified phosphorylation-site localization workflow. By harmonizing algorithm inputs and outputs and implementing a standardized alanine-decoy strategy, onsite supports empirical estimation and consistent control of the global false localization rate across multiple LC–MS/MS files.

Independent benchmarking against the known phosphorylation sites in the synthetic PXD000138 data set confirmed the localization performance of the integrated algorithms while revealing distinct trade-offs between yield and accuracy. At a 5% global FLR, pyLucXor recovered the largest number of correctly localized sites, whereas AScore and PhosphoRS achieved slightly higher localization accuracy. Analysis of the large-scale PXD012255 data set further revealed substantial agreement among the algorithms together with sizable method-specific site sets, demonstrating their complementary contributions to phosphosite coverage.

Implemented using PyOpenMS and distributed via PyPI and BioContainers, onsite can be deployed as a standalone tool or as part of the quantms workflow. Its containerized implementation supports reproducible execution across local workstations, high-performance computing systems, and cloud environments. Together, standardized global FLR estimation, independent ground-truth validation, and scalable workflow integration make onsite a practical framework for reproducible, cross-algorithm comparable phosphorylation-site localization in large-scale phosphoproteomics. Future work will focus on improving computational efficiency and extending the framework to additional post-translational modifications.

## Supplementary Material



## Data Availability

onsite is an open-source Python library (https://github.com/bigbio/onsite). All data sets reanalyzed in this study are publicly available in the PRIDE repository under accession numbers PXD000138 and PXD012255.

## Notes

Search and workflow parameters for PXD000138 and PXD012255 (Table S1); search and workflow parameters for PXD000138 and PXD012255 (Table S2); global pipeline parameters (Table S3); computational time benchmark and throughput for the evaluated algorithms integrated within the onsite toolkit (Table S4); pyLucXor-specific parameters (Table S5); orthogonal analysis for all phosphosite intersections (Table S6); sequence motif analysis of consensus and pyLucXor-specific phosphosites (Figure S1); and unique phospho sites at different FLR thresholds measured on the synthetic ground-truth data set (Figure S2) (PDF)
