# Supplementary material for: onsite: An Integrated Framework for Phosphosite Localization and False Localization Rate Estimation
Source: J Proteome Res. 2026 Oct 10;25(11):6507–13. doi: 10.1021/acs.jproteome.6c00570 (PMC13750069; doi:10.1021/acs.jproteome.6c00570)

Supporting information

onsite: An Integrated Framework for Phosphosite Localization and False Localization Rate Estimation

Qi-Xuan Yue [a], Zhongchun Wei [a], Chengxin Dai [b,c], Mingze Bai [a], Yasset Perez-Riverol [d], Timo Sachsenberg [e,f*]

*Corresponding author: Timo Sachsenberg (timo.sachsenberg@uni-tuebingen.de)

a. Chongqing Key Laboratory of Big Data for Bio Intelligence, Chongqing University of Posts and Telecommunications, Chongqing, China

b. State Key Laboratory of Medical Proteomics, Beijing Proteome Research Center, National Center for Protein Sciences (Beijing), Beijing Institute of Lifeomics, 102206, Beijing, China

c. International Academy of Phronesis Medicine (Guangdong), 510320, Guangdong, China

d. European Molecular Biology Laboratory, European Bioinformatics Institute, Wellcome Genome Campus, Cambridge, United Kingdom

e. Applied Bioinformatics, Department of Computer Science, University of Tübingen, Tübingen, Germany

f. Institute for Bioinformatics and Medical Informatics, University of Tübingen, Tübingen, Germany

Content

Supplementary Table S1: Search and workflow parameters for PXD000138 and PXD012255. ...2

Supplementary Table S2: Commands and parameters of the evaluated algorithms. 3

Supplementary Table S3: Global pipeline parameters (these parameters are common to all three algorithms). 3

Supplementary Table S4: Computational time benchmark and throughput for the evaluated algorithms integrated within the onsite toolkit. 4

Supplementary Table S5: pyLucXor specific parameters. 5

Supplementary Table S6: Orthogonal analysis for all phosphosite intersections in Figure 2B. For each algorithmic intersection, the table reports the total number of localized sites, the number and percentage of sites independently validated in the external reference resource (PeptideAtlas), the average external observation count, and the average number of supporting PSMs and fractions within the PXD012255 dataset. 6

Supplementary Figure S1: Sequence motif analysis of consensus and pyLucXor-specific phosphosites (PXD012255). 7

Supplementary Figure S2: Unique Phospho sites at different FLR thresholds measured on the synthetic ground-truth dataset (PXD000138). 8

Supplementary Table S1: Search and workflow parameters for PXD000138 and PXD012255.

Category	Configuration Parameter	PXD000138	PXD012255
Workflow Versions	quantms pipeline version	v1.3.1dev	v1.8.0dev
	Nextflow version	24.04.4	25.10.4
Search Engines	Comet version	2023.01 rev. 2	2025.01 rev. 1
	MS-GF+ version	v2023.01.12	v2024.03.26
Database	Reference Proteome	Homo sapiens	Homo sapiens
Cleavage Rules	Enzyme / Cleavage agent	Trypsin/P	Trypsin
	Allowed missed cleavages	2	2
Mass Tolerances	Precursor mass tolerance	5 ppm	20 ppm
	Fragment mass tolerance	0.02 Da	0.02 Da
Modifications	Fixed modifications	-	Carbamidomethyl (C)
	Variable modifications	Phospho (S,T,Y), Oxidation (M)	Phospho (S,T,Y), Oxidation (M), Acetyl (Protein N-term)
	Max variable mods per peptide	3	3
Error Control	Method	Percolator (v3.05.0)	Percolator (v3.06.0)
	PSM-level FDR (q-value) threshold	0.01	0.01

Supplementary Table S2: Commands and parameters of the evaluated algorithms.

Algorithm	Commands and parameters
AScore (onsite)	onsite ascore -in mzml_path -id idxml_path -out output_path -threads 1 --fragment-mass-tolerance 0.05 --add-decoys
PhosphoRS (onsite)	onsite phosphors -in mzml_path -id idxml_path -out output_path -threads 1 --fragment-mass-tolerance 0.05 --add-decoys
pyLucXor (onsite)	onsite lucxor -in mzml_path -id idxml_path -out output_path -threads 1 --fragment-method HCD --fragment-mass-tolerance 0.05 --target-modifications "Phospho(S),Phospho(T),Phospho(Y),PhosphoDecoy(A)"
Luciphor (original)	LuciphorAdapter -in mzml_path -id idxml_path -out output_path -executable /path/to/luciphor2.jar -target_modifications 'Phospho (S)' 'Phospho (T)' 'Phospho (Y)' -fragment_mass_tolerance 0.05 -fragment_error_units Da -fragment_method HCD -fragment_error_value 0.05 -fragment_error_unit Da -threads 1 -num_threads 1

Luciphor: quay.io/biocontainers/openms-thirdparty:3.5.0--h9ee0642_0

Supplementary Table S3: Global pipeline parameters (these parameters are common to all three algorithms).

Parameter	Description	Default value
-in	Input spectrum file	mzML
-id	Input identification file	MzIdentML, idXML, idparquet (QPX)
-out	Output file	-
--fragment-mass-tolerance	Fragment mass tolerance for the global search	0.05
--fragment-mass-unit	Unit for fragment mass tolerance	Da
--threads	Number of parallel threads for execution	1
--add-decoys	Add decoy modifications	False

--compute-all-scores	Run all three localization algorithms and merge the results	-

Supplementary Table S4: Computational time benchmark and throughput for the evaluated algorithms integrated within the onsite toolkit.

Algorithm	Execution Time (seconds)	Throughput (PSMs/second)
Luciphor (original)	5426.65	62.10
pyLucXor (onsite)	5078.61	66.36
AScore (onsite)	1483.43	227.17
PhosphoRS (onsite)	1504.07	224.05

Note: The benchmark was performed on a workstation with an Intel Core i9-7900X CPU, 128 GB RAM, and Ubuntu 24.04.2 LTS.

Supplementary Table S5: pyLucXor specific parameters.

Parameter	Description	Default value
--fragment-method	Fragmentation method utilized (CID or HCD)	CID
--fragment-mass-tolerance	Tolerance for matching fragment ions	0.5
--min-mz	Minimum m/z threshold; peaks below this value are ignored	150.0
--target-modifications	Target modification residues	'Phospho(S),Phospho(T),Phospho(Y)'
--neutral-losses	List of target neutral losses	sty -H3PO4 -97.97690
--decoy-mass	Mass added for decoy modification generation	79.966331
--decoy-neutral-losses	List of decoy neutral losses	X -H3PO4 -97.97690
--max-charge-state	Maximum precursor charge state to consider	5
--max-peptide-length	Maximum allowed peptide length	40
--max-num-perm	Maximum number of permutations	16384
--modeling-score-threshold	Threshold applied for score modeling	0.95
--scoring-threshold	Minimum score threshold	0.0
--min-num-psms-model	Minimum number of PSMs for modeling	50
--seed	Random Number Generator (RNG) seed for reproducible decoy permutations and model subsampling	42
--rt-tolerance	Retention time tolerance	0.01
--disable-split-by-charge	Disable the splitting of scoring models	False

	by charge state	

Supplementary Table S6: Orthogonal analysis for all phosphosite intersections in Figure 2B. For each algorithmic intersection, the table reports the total number of localized sites, the number and percentage of sites independently validated in the external reference resource (PeptideAtlas), the average external observation count, and the average number of supporting PSMs and fractions within the PXD012255 dataset.

Intersection	Total sites	Validated sites	Validation percentage	PeptideAtlas observation (mean)	PXD012255 Supporting PSMs (mean)	PXD012255 Fractions (mean)
pyLucXor exclusive	3742	3450	92.20%	1240.5	1.91	1.23
PhosphoRS exclusive	355	291	81.97%	1278.9	1.21	1.06
AScore exclusive	177	149	84.18%	1327.8	1.14	1.06
pyLucXor & PhosphoRS	1164	1070	91.92%	2336	3.61	1.53
pyLucXor & AScore	414	380	91.79%	2334.4	3.75	1.58
AScore & PhosphoRS	251	188	74.90%	1846.4	2.29	1.17
Shared by All	4421	4092	92.56%	3108	8.36	1.86

Supplementary Figure S1: Sequence motif analysis of consensus and pyLucXor-specific phosphosites (PXD012255).

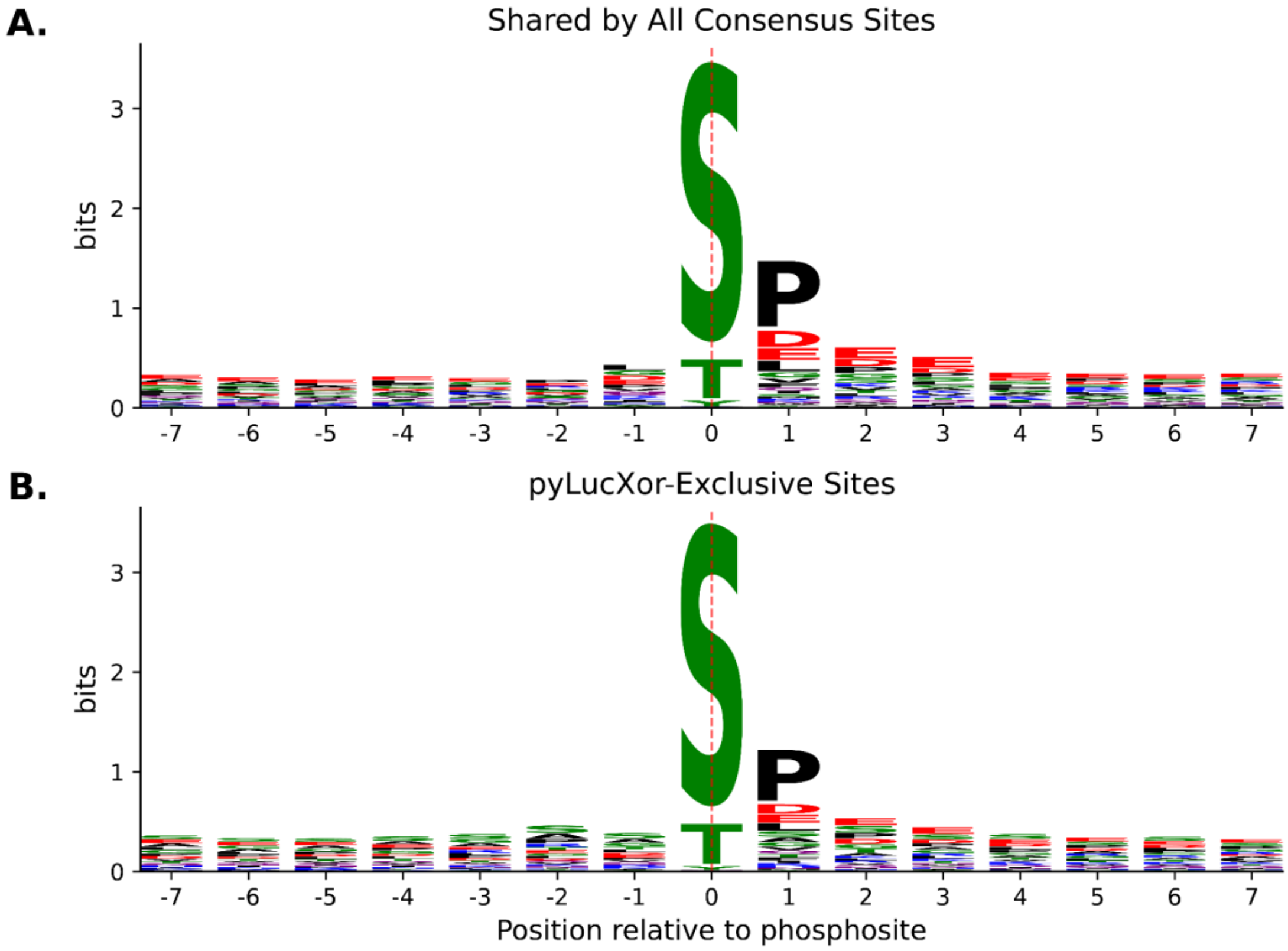

Supplementary Figure S2: Unique Phospho sites at different FLR thresholds measured on the synthetic ground-truth dataset (PXD000138).

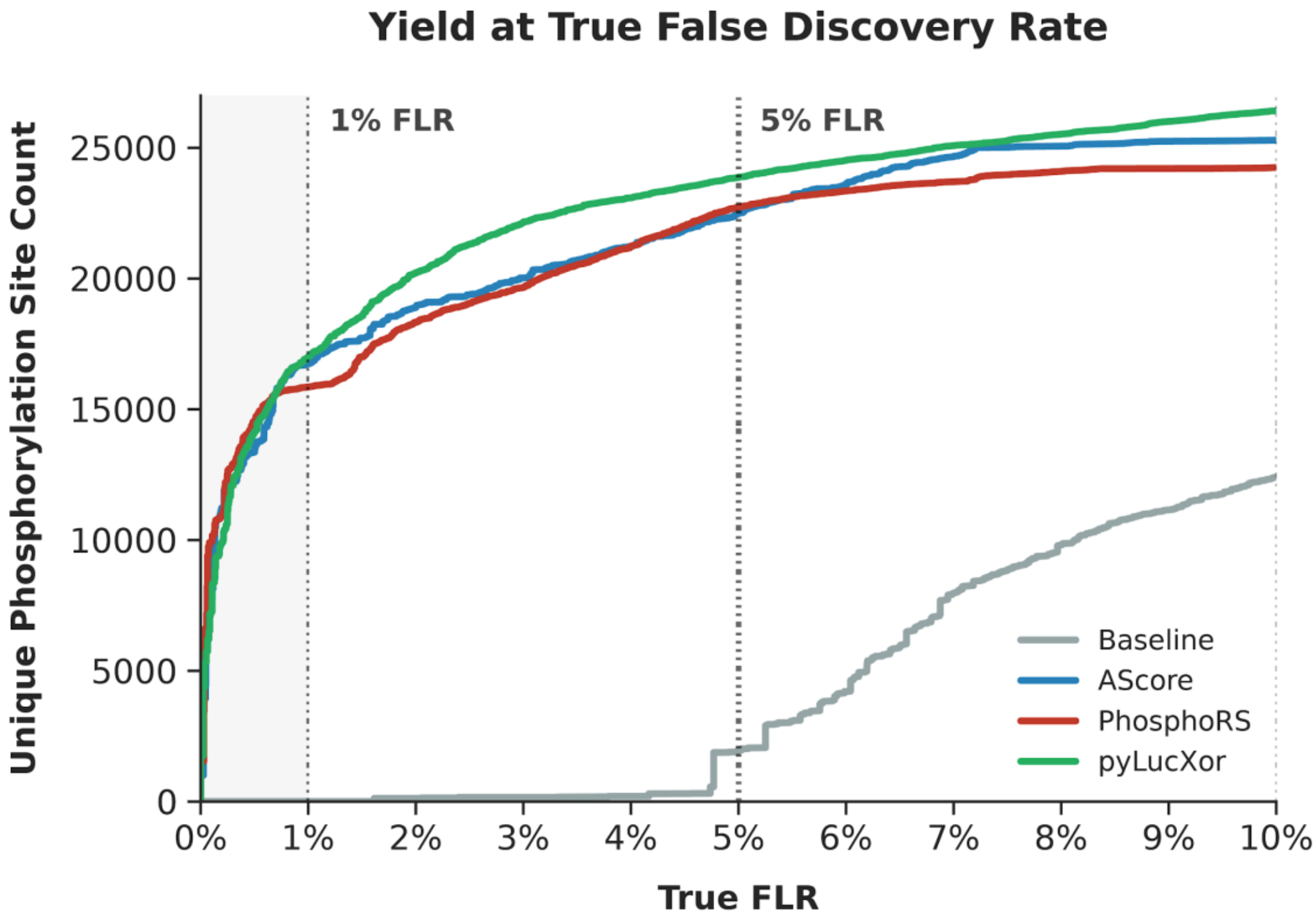

Supplement: Supplementary file 1 [file pr6c00570_si_001.pdf]
